# Application of Force to a Syndecan-4 Containing Complex With Thy-1–α_V_β_3_ Integrin Accelerates Neurite Retraction

**DOI:** 10.3389/fmolb.2020.582257

**Published:** 2020-09-29

**Authors:** Francesca Burgos-Bravo, Samuel Martínez-Meza, Andrew F. G. Quest, Christian A. M. Wilson, Lisette Leyton

**Affiliations:** ^1^Laboratory of Cellular Communication, Center for Studies on Exercise, Metabolism and Cancer, Institute of Biomedical Sciences, Santiago, Chile; ^2^Advanced Center for Chronic Diseases, Facultad de Medicina, Universidad de Chile, Santiago, Chile; ^3^Single Molecule Biochemistry and Mechanobiology Laboratory, Department of Biochemistry and Molecular Biology, Facultad de Ciencias Químicas y Farmacéuticas, Universidad de Chile, Santiago, Chile

**Keywords:** cell–cell adhesion, single-molecule analysis, trimolecular adhesion complex, mechano-sensor, mechano-transduction, inflammation, cell adhesion molecules

## Abstract

Inflammation contributes to the genesis and progression of chronic diseases, such as cancer and neurodegeneration. Upregulation of integrins in astrocytes during inflammation induces neurite retraction by binding to the neuronal protein Thy-1, also known as CD90. Additionally, Thy-1 alters astrocyte contractility and movement by binding to the mechano-sensors α_V_β_3_ integrin and Syndecan-4. However, the contribution of Syndecan-4 to neurite shortening following Thy-1–α_V_β_3_ integrin interaction remains unknown. To further characterize the contribution of Syndecan-4 in Thy-1-dependent neurite outgrowth inhibition and neurite retraction, cell-based assays under pro-inflammatory conditions were performed. In addition, using Optical Tweezers, we studied single-molecule binding properties between these proteins, and their mechanical responses. Syndecan-4 increased the lifetime of Thy-1–α_V_β_3_ integrin binding by interacting directly with Thy-1 and forming a ternary complex (Thy-1–α_V_β_3_ integrin + Syndecan-4). Under *in vitro*-generated pro-inflammatory conditions, Syndecan-4 accelerated the effect of integrin–engaged Thy-1 by forming this ternary complex, leading to faster neurite retraction and the inhibition of neurite outgrowth. Thus, Syndecan-4 controls neurite cytoskeleton contractility by modulating α_V_β_3_ integrin mechano-receptor function. These results suggest that mechano-transduction, cell-matrix and cell-cell interactions are likely critical events in inflammation-related disease development.

## Introduction

Cell–cell adhesion is initiated by the interaction in “trans” of membrane receptors located on opposing cell surfaces. An example of such heterophilic interactions is that mediated by the glycosyl-phosphatidylinositol (GPI)-anchored adhesion protein Thy-1 (expressed on many cells including neurons, fibroblasts, thymocytes and cancer cells) with integrins, such as α_V_β_3_ on astrocyte and α_5_β_1_ on human melanoma cells (Herrera-Molina et al., 2013). These transmembrane mechano-receptors are well known to connect extracellular matrix ligands to the cytoskeleton to promote cell adhesion, contractility, and migration. However, association also occurs between oligomerized, preassembled Thy-1 complexes and integrin clusters located on adjacent cell surfaces (Hagood, 2019; Leyton et al., 2019). For example, under inflammatory conditions in the CNS, α_V_β_3_ integrin clustering, and the intracellular signaling cascade initiated in astrocytes by neuronal Thy-1 binding, leads to the assembly of focal adhesions (FA) and cell motility (Kong et al., 2013; Lagos-Cabré et al., 2017). Alternatively, α_V_β_3_ integrin binding to Thy-1 complexes on an adjacent cell (the neuron), triggers neurite retraction or inhibition of neurite outgrowth (Herrera-Molina et al., 2012; Maldonado et al., 2017). Another example is the binding of melanoma integrins to the Thy-1-expressing cytokine-activated endothelium, which increases endothelial cell contractility (Schubert et al., 2013). In this case, cell contraction results in extravasation of cancer cells through the blood vessel wall, an important event for establishing secondary tumors (Bendas and Borsig, 2012). These examples underscore the importance of studying the effect of forces generated by alterations in cytoskeletal tension on the cell responses mediated by Thy-1–integrin association.

Bonds supporting protein-protein interactions respond to mechanical forces in three manners, force might (i) accelerate bond dissociation (slip bond); (ii) slow down dissociation (catch bond); or (iii) maintain bond lifetime (ideal bond) (Dembo et al., 1988; Zhu, 2014). Molecular force spectroscopy is a technique employed to characterize the effect of force on these interactions, and is used to obtain information concerning bond strength, the energy landscape of the dissociation process and the lifetime of bimolecular interactions (Yuan et al., 2000; Stangner et al., 2013).

In a recent study, binding of Thy-1-coated beads to the purified α_5_β_1_ integrin ectodomain was shown to exhibit a slip bond behavior (non-stiffening bond). Additionally, when the Thy-1-beads were challenged with α_5_β_1_ integrin expressed in K562 cells, a “dynamic catch” behavior was observed, that is the slip bond changed to a catch bond (stiff bond) and then reverted back to slip bond behavior, as the force applied increased (Fiore et al., 2014). In these experiments, antibodies against α_5_β_1_ integrin only partially blocked the interactions, suggesting the participation of other adhesive components in the bead-cell model. This led to the discovery of the heparan sulfate proteoglycan Syndecan-4 as the missing protein component that, by forming a tri-molecular complex with Thy-1 and the integrin, changes the stiffness of the bonds involved under mechanical stimulation.

Interestingly, the bi-directional signaling described by our group studying neuron-astrocyte interactions under pro-inflammatory conditions, induced for instance by traumatic brain injury or neurodegenerative diseases was shown to require similar components. That is, Thy-1 binding to α_V_β_3_ integrin in astrocytes requires Syndecan-4 to induce FA turnover and forward cell motility in reactive astrocytes (Avalos et al., 2009; Kong et al., 2013). Thy-1-stimulated astrocyte migration is an essential response of this glial cell to reach and repair the damage zone (Lagos-Cabré et al., 2017). Alternatively, α_V_β_3_ integrin binding to Thy-1 on neurons leads to neurite retraction (Herrera-Molina et al., 2012; Maldonado et al., 2017), an early response of axons to injury required to remove at a small-scale damaged axonal connections, to stabilize the affected neuronal network and to initiate the repairing processes (Houle and Tessler, 2003; Luo and O’Leary, 2005). However, a role for Syndecan-4 in the α_V_β_3_ integrin-induced alteration of the neuronal actin-myosin cytoskeleton has not been reported.

Thus, the physical properties and the biological significance underlying the formation of the tri-molecular complex in neuron-astrocyte interactions were studied. To this end, bond behavior under force was analyzed using optical miniTweezers in combination with various cell-based assays. Here, we report that Thy-1–α_V_β_3_ integrin binding, and the neuronal responses elicited downstream of this interaction are modulated by Syndecan-4 association. In addition, and contrary to the dynamic catch model previously reported (Fiore et al., 2014), we found that Syndecan-4 does not change the slip bond properties of the Thy-1–α_V_β_3_ integrin interaction, but rather stabilizes the interaction between these two proteins, thereby potentiating the integrin effect in neurons. Importantly, only reactive astrocytes induced these outcomes in neurons, suggesting that inflammatory conditions are required to favor the ternary complex formation. These studies yield important insights to how inflammation affects neuronal architecture inducing changes in mechano-transduction that will contribute to neurodegeneration.

## Materials and Methods

### Antibodies and Reagents

Immunoblot analysis of astrocyte cell lysates was performed using antibodies, including anti-β_3_ integrin (AB2984; Millipore), anti-Δ-heparan sulfate (3G10; Seikagaku), anti β actin (A5060; Sigma-Aldrich), and secondary horseradish peroxidase-conjugated goat anti-mouse (074-1806) or anti-rabbit (074-1516) IgG polyclonal secondary antibodies from KPL. Anti-Syndecan-4 (sc-12766; Santa Cruz) was used to detect the recombinant Syndecan-4-Fc. Goat polyclonal antibody anti-rat β_3_ integrin (sc-6627; Santa Cruz) and rabbit polyclonal anti-rat β_1_ integrin (sc-8978; Santa Cruz) were used as blocking antibodies in the co-culture assays. Reagents used in this work were: Heparin (H-3400; Sigma-Aldrich), Heparitinase III from *Flavobacterium heparinum* EC 4.2.2.8 (H-8891; Sigma-Aldrich), Cell TrackerTM Green 5-chloromethyl fluorescein diacetate dye (C2925; Invitrogen) for monitoring living cells. The cell Line Nucleofector Kit (VCA-1003; Lonza) was used to transfect the astrocytes in the Nucleofector Device, X-treme GENE HP DNA Transfection Reagent (06-366 244 01; Roche) for HEK293T cell transfection, 1-Step Ultra TMB (3,3′,5,5′-tetramethylbenzidine) ELISA substrate (34028; Thermo Scientific), recombinant human bFGF protein (PH-G0266; Gibco), bovine serum albumin fraction V protease- and immunoglobulin-free (BSA-50; Rockland), protein A-sepharose from *Staphylococcus aureus* (P-3391; Sigma) and protein G-polystyrene beads (3.1 and 2.1 μm; Spherotech).

### Cell Cultures

CAD cells (Cath.a-differentiated) were used as a neuronal model to study neuronal process outgrowth (Qi et al., 1997; Li et al., 2007). CAD cells were grown in DMEM/F12 medium (Gibco, United States) supplemented with 8% of fetal bovine serum (FBS HyClone, Canada) and morphological and functional differentiation of CAD cells was induced by serum deprivation for 24 h in DMEM/F12 supplemented with 50 ng/ml of sodium selenite (S5261; Sigma-Aldrich) as reported (Herrera-Molina et al., 2012). The astrocyte cell line DITNC1 was maintained in RPMI 1640 medium (Gibco) with 5% FBS (HyClone, United States) and 0.1 mM 2-mercaptoethanol (Gibco). Primary astrocytes were derived from mixed glial cell cultures recovered from cortices of 2-day-old rats (P2) (bioethical protocol approved by the biothical committe of the Universidad de Chile) and cultured with DMEM/F12 medium supplemented with 10% FBS (Biological Industries) as previously described (Lagos-Cabré et al., 2017). HEK293T cells used to produce recombinant α_V_β_3_-Fc and Syndecan-4-Fc proteins were grown in DMEM/High-glucose medium supplemented with 10% FBS (Hyclone, Canada). All cells were maintained with 1% penicillin-streptomycin solution on standard tissue culture dishes in a humidified atmosphere of 5% CO_2_ and 37°C.

### Recombinant Fc-Tagged Proteins

Purified Thy-1-Fc wild-type, Thy-1(RLE)-Fc mutant for the integrin-binding site, Thy-1(AEAAA)-Fc mutant for the HBD, as well as human TRAIL-R2-Fc fusion proteins were obtained as previously reported (Schneider, 2000; Leyton et al., 2001). Recombinant α_V_β_3_-Fc integrin, possessing the ectodomain of the heterodimeric protein and the Fc portion of the human immunoglobulin IgG1, was secreted into serum-free cell culture media of transiently transfected HEK293T cells and purified as previously published (Burgos-Bravo et al., 2018). A similar experimental strategy was used to produce the Fc-tagged protein of Syndecan-4 (Syndecan-4-Fc), where the extracellular domain of the human proteoglycan at the C terminus was fused to the Fc tag. Here, HEK293T cells were transfected with the Syndecan-4-Fc expression plasmid using the X-treme GENE HP DNA transfection reagent according to the manufacturer’s instructions (Roche). After 2 days in culture, serum-free medium containing soluble Syndecan-4-Fc was recovered, filtered and stored at -20°C. Commercially purified human Syndecan (ectodomain)-4-Fc was used for optical tweezers experiments.

### Characterization of Syndecan-4-Fc Functionality

HS chains on the recombinant Syndecan-4-Fc protein were characterized by the electrophoretic mobility of Syndecan-4-Fc after treatment with Heparitinase (Hase III), which removes the HS chains from the core protein. Syndecan-4-Fc was first precipitated from the serum-free medium obtained from HEK293T transfected cells, by incubating for 1 h at 4°C, with an excess of protein A-sepharose beads. Then, the solution was centrifuged (3000 *g* × 5 min) and the precipitate contained the Syndecan-4-Fc protein (Precipitated; Figure 2B), while the medium was depleted of the fusion protein (Depleted; Figure 2B). All samples were digested for 3 h at 37°C with 0.5 mU Hase III and resuspended in the digestion buffer (20 mM Tris-HCl, pH 7.4 containing 50 mM NaCl and 2 mM CaCl_2_). As controls, undigested samples were also prepared by incubating them only with the digestion buffer. Samples were then boiled for 5 min in Laemmli buffer (2% SDS, 10% Glycerol, 62.5 mM Tris-HCl, pH 6.8, 5% β-mercaptoethanol and 0.01% bromophenol blue), separated by 10% SDS-PAGE gels, transferred onto nitrocellulose membrane (Millipore) and blocked in 5% w/v non-fat, dry milk in TBS containing 0.1% Tween-20. Immunoblots were analyzed by incubation of membranes with anti-Syndecan-4 antibodies (1:2000, Santa Cruz) for 1 h at room temperature. Membranes were then washed and incubated with horseradish peroxidase-conjugated goat anti-mouse IgG (1:3000, KPL) for 1 h at room temperature. The peroxidase activity was revealed with a chemiluminescence kit (Pierce, Thermo Scientific). The functionality of Syndecan-4-Fc was tested by enzyme-linked immunosorbent assay (ELISA). First, bFGF (1 μg/ml) or BSA (1 μg/ml; control) were immobilized overnight in a 96-well plate (Maxisorp, Nunc) at 4°C. Then, serum-free medium containing Syndecan-4-Fc protein as well as TRAIL-R2-Fc protein (Fc-tagged protein control) were added to wells and incubated for 2 h at 37°C. To detect Syndecan-4-Fc/bFGF binding, HRP-coupled goat anti-human IgG1 antibodies and the chromogenic TMB substrate were used according to manufacturer’s instructions (Thermo Scientific).

### Neurite Outgrowth Assay

DITNC1 cells (5 × 10^5^ cells/cm^2^) or primary astrocytes (3 × 10^5^ cells/cm^2^) were seeded and grown to 90% confluency on 8-well glass bottom plates. To induce a pro-inflammatory environment, rat primary astrocytes were stimulated with 10 ng/ml of TNF for 48 h. Astrocyte monolayers were treated with Hase III (0.5 mU) for 3 h at 37°C in serum-free medium to study whether heparin sulfate chains were involved in neurite outgrowth inhibition. In other experiments, astrocyte Syndecan-4 was silenced using siRNA against exons 3 and 4, as reported (Avalos et al., 2009). Control siRNA (siCTRL) was used to confirm specificity. Astrocytes were fixed with 4% p-formaldehyde for 30 min at room temperature and washed with abundant PBS containing 5 mM glycine. Then, fixed monolayers were incubated with anti-β_3_ integrin antibody (5 μg/ml) during 1 h at 37°C to block β_3_ or β_1_-containing heterodimeric integrins in the astrocyte surface, respectively. Next, cell tracker green-labeled CAD cells (5 × 10^4^ cells/cm^2^) were added to the astrocyte monolayer and maintained in co-culture for 24 h. In some experiments, CAD cells were pre-incubated with Heparin (400 μg/ml) for 30 min at room temperature to block astrocyte surface HS chain binding. The next day, cells were washed with PBS and morphological differentiation of CAD cells (i.e., neurite outgrowth) was induced in serum-free medium for 24 h. Images of living cells were acquired using a FV10i confocal microscope (Olympus Corp., Tokyo, Japan) equipped with UPLSAPO 60X/1.35 water immersion objective. CAD cells were analyzed using NeuronJ plug-in for ImageJ software as was previously reported (Herrera-Molina et al., 2012; Maldonado et al., 2017). The length of processes was measured and expressed in μm. CAD cells were considered morphologically differentiated when presenting at least one neurite longer or equal to 15 μm. The number of varicosities per neuronal process was also used to characterize differentiated CAD cells.

### Neurite Retraction Assay

CAD cells (5 × 10^4^ cells/cm^2^) were seeded in 24-well plastic plates and grown in serum-containing DMEME/F12 medium overnight to reach 60% confluency. Then, morphological differentiation was induced in serum-free medium for 24 h as indicated before. CAD cells with established neurites (i.e., with neuronal processes) were incubated with serum-free medium containing α_V_β_3_-Fc integrin (one tenth of the total volume), in the absence or presence of different volumes of Syndecan-4-Fc-containing medium at 37°C. Control cells were incubated with Fc-tagged depleted medium, which was obtained by overnight incubation with an excess of protein A-sepharose beads. In other experiments, medium-containing α_V_β_3_-Fc protein was pre-incubated with Protein-A (2 μg) and added to morphologically differentiated CAD cells. Neurite length changes were recorded after 5, 10, 20, and 40 min of exposure to Syndecan-4-Fc and/or α_V_β_3_-Fc-containing supernatant using a Disk Scanning Unit-IX81 Spinning disk confocal microscope (Olympus Corp.). The neuronJ plug-in was used to quantify neurite length over time.

### Immunoblotting Analysis

For immunoblot analysis of DITNC1 cells or primary astrocytes, cells were washed with ice-cold PBS and lysed with ice-cold lysis buffer (150 mM NaCl, 0.1% SDS, 0.25% sodium deoxycholate,1% Triton-X100, in 50 mM Tris-HCl pH, 7.0) supplemented with protease and phosphatase inhibitors (1 mM sodium orthovanadate, 2 μg/ml antipain, 1 μg/ml leupeptin, 10 μg/ml benzamidine and 1 mM PMSF). Protein extracts (30 μg) were mixed with Laemmli buffer, boiled for 5 min, electrophoretically separated on 10% SDS-PAGE and transferred to nitrocellulose membranes (Millipore). For Syndecan-4 detection, protein extracts were first digested with Heparitinase III (0.5 mU) for 3 h at 37°C and then mixed with Laemmli buffer. The membranes were blocked with TBS-T 5% fat-free milk and subsequently incubated with anti-Δ-heparan sulfate (1:2500; Seikagaku), anti-β_3_ integrin (1:3000; Millipore), or anti-β-actin (1:3000; Sigma-Aldrich) antibodies for 1 h at room temperature, followed by the appropriate horseradish peroxidase-conjugated secondary antibodies. In all cases, peroxidase activity was revealed with the chemiluminescence kit (Pierce, Thermo Scientific). Immunoblot quantification was performed by measuring band intensity using ImageJ software and normalized to the loading control (β actin).

### Optical Tweezers and Force Measurement Protocols

**FIGURE 1:**
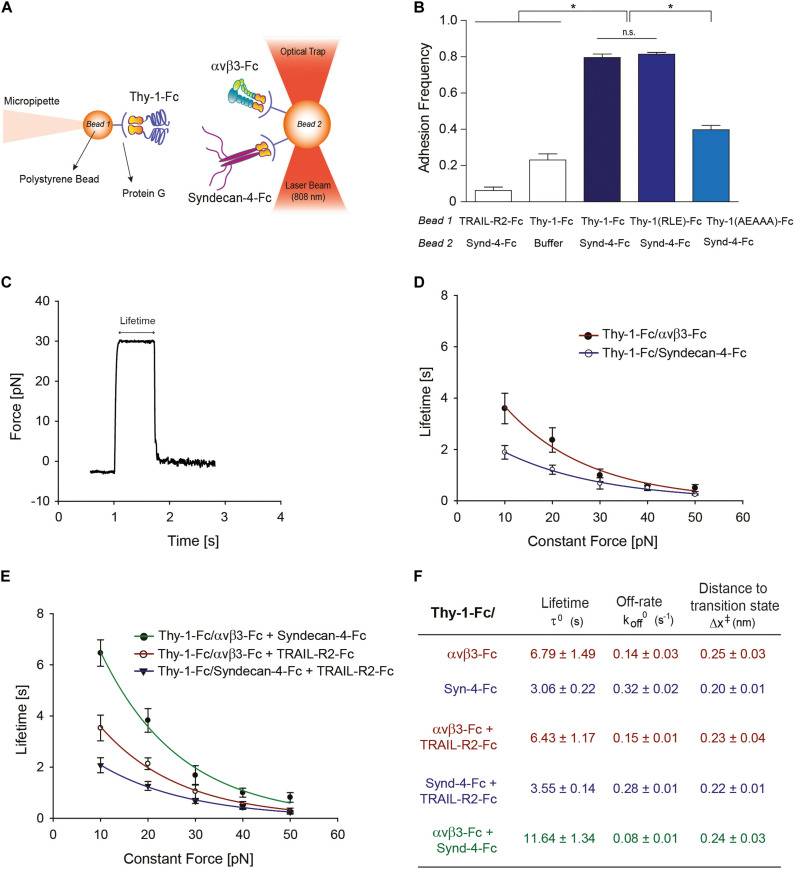
Characterization at the single-molecule level reveals that Syndecan-4 increases the lifetime of the Thy-1–α_V_β_3_ integrin binding by forming a ternary complex. **(A)** Scheme of the assay using miniTweezers. Two different sizes of protein G-coated beads were used; the smaller bead (Bead 1; 2.1 μm) contained purified Thy-1-Fc and was attached to a micropipette by suction; the larger bead (Bead 2; 3.1 μm) containing the α_V_β_3_-Fc, Syndecan-4-Fc or both molecules, was trapped by a laser beam and held in the focus of the microscope (Figure adapted from Burgos-Bravo et al., 2018. https://doi.org/10.1091/mbc.E17-03-0133). **(B)** Adhesion frequency of Syndecan-4 (Synd-4-Fc) with TRAIL-R2-Fc (control protein), wild-type Thy-1-Fc, Thy-1(RLE)-Fc mutated in the integrin binding-site, and Thy-1-(AEAAA)-Fc mutated in the heparin binding domain, was assessed using force-ramp assays at a loading rate of 10 pN/s. The total number of binding events in at least 50 approaching-retraction cycles per 4–5 pairs of beads were measured. Non-specific interactions were evaluated using Thy-1-Fc- and Hepes buffer-treated beads (Buffer). Data are expressed as the mean ± SEM (*p < 0.05; n.s. non-significant, assessed by Mann–Whitney’s test). **(C)** Representative force-time trace obtained by force-constant assay at 30 pN between Thy-1-Fc and Syndecan-4-Fc. The force is ramped to and sustained at a constant force until the interaction is disrupted. **(D)** Lifetime of bi-molecular Thy-1-Fc interactions with Syndecan-4-Fc (Thy-1-Fc/Syndecan-4-Fc) or α_V_β_3_-Fc integrin (Thy-1-Fc/α_V_β_3_-Fc) as a function of the constant force. **(E)** Lifetime of tri-molecular Thy-1-Fc interactions with α_V_β_3_-Fc integrin and Syndecan-4-Fc (Thy-1-Fc/α_V_β_3_-Fc + Syndecan-4-Fc) plotted versus constant force. As a control for the tri-molecular interactions, lifetime data were evaluated for Thy-1-Fc binding with TRAIL-R2-Fc in the presence of Syndecan-4-Fc (Thy-1-Fc/Syndecan-4-Fc + TRAIL-R2-Fc) or α_V_β_3_-Fc integrin (Thy-1-Fc/α_V_β_3_-Fc + TRAIL-R2-Fc). Lifetime data plotted against constant forces were fitted to the Bell model (see Materials and Methods) to calculate the unbinding parameters at zero force for each interaction **(F)**, including lifetime (τ^0^), off-rate constants (k_off_^0^; inversely related to the lifetime), and the distance to the transition state (Δx^‡^). Lifetime data in **(D,E)** are expressed as the mean ± SEM from at least 60 binding events obtained using 3 pairs of different beads. Force-ramp and constant-force results were analyzed by a Matlab program.

To characterize the mechanical properties of the Thy-1-dependent interactions, a miniTweezers device was used as a single-molecule force transducer instrument (Smith et al., 2003), using a stiffness of 0.1 pN/nm. All binding experiments were performed in Hepes buffer (10 mM Hepes, pH 7.4) containing 100 mM NaCl and 1 mM MgCl_2_ at 25°C, using a microchamber with a glass micropipette as reported (Burgos-Bravo et al., 2018). Protein G-coated polystyrene beads were used to attach the Fc-tagged proteins at a femtomolar concentration (description in Figure 1A). In order to characterize the rupture forces required to dissociate the Thy-1-Fc-dependent interactions and determine the adhesion frequency between Thy-1-Fc (wild-type and mutants) and Syndecan-4-Fc, a force-ramp assay was performed pulling the optically trapped bead at a constant force-loading rate (10 pN/s) as described (Burgos-Bravo et al., 2018). The rupture force histograms were obtained with at least 120 binding events per four pairs of freshly prepared beads and normalized for the total number of approaching-retraction cycles (i.e., when both protein-coated beads are put in contact to promote the protein-protein interaction and then are separated at a constant rate to dissociate the binding). These histograms contain rupture forces associated to non-specific binding events of Thy-1-Fc (Figure 1B, Thy-1/buffer condition), however, for comparison studies, we assumed that these non-specific interactions of lower affinity were affecting each histogram in similar magnitude, mainly in the low range of force values. The bin size was calculated using Scott’s rule (Scott, 1979). For the adhesion frequency assay, events with and without binding were characterized to calculate an adhesion frequency in at least 50 approach-retraction cycles carried out per 4–5 pairs of different beads. The lifetime of each bond (Thy-1-Fc–Syndecan-4-Fc, Thy-1-Fc–α_V_β_3_-Fc or Thy-1–α_V_β_3_-Fc + Syndecan-4-Fc) was determined using the constant-force assay, in which the optically trapped bead is placed in close proximity of the other bead to promote bond formation and then quickly subjected to a ramp force (<1 s) at a constant value (constant forces applied here were: 10, 20, 30, 40, and 50 pN) until the interaction is dissociated. The bond lifetime was measured as the time from when the force reached the desired level until the instant the binding was disrupted (Figure 1C). At each constant force condition, a collection of lifetime data was obtained and plotted as a function of the constant force. The Bell model:

**FIGURE 2:**
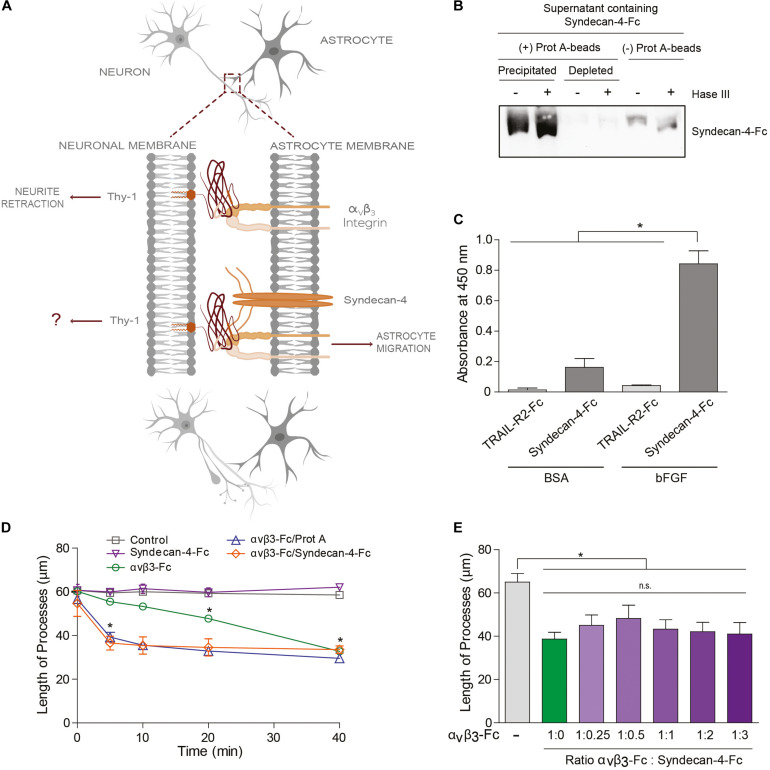
Syndecan-4 accelerates neurite retraction promoted by the α_V_β_3_ integrin. **(A)** Thy-1–α_V_β_3_ integrin binding induces cell signaling events resulting in the retraction of neuronal processes. On the other hand, Thy-1–α_V_β_3_ integrin + Syndecan-4 association promotes astrocyte migration. Here, the effect of Syndecan-4 on α_V_β_3_ integrin-induced neurite retraction was tested. **(B)** Serum-free medium containing Syndecan-4-Fc fusion protein was incubated (+) or not (–) with an excess of protein-A-sepharose beads and then centrifuged to obtain a precipitated Syndecan-4-Fc-protein-A-sepharose complex and Syndecan-4-depleted supernatant, respectively. All these samples were treated (+) or not (–) with Heparitinase III, separated by SDS-PAGE and analyzed by immunoblotting with anti-Syndecan-4 antibodies. **(C)** A microplate coated with human bFGF (1 μg/ml) or BSA (1 μg/ml) was incubated with serum-free medium containing Syndecan-4-Fc or TRAIL-R2-Fc (control Fc-protein), followed by incubation with anti-Fc-HRP conjugated antibody. Specific binding was measured in a colorimetric method with TMB substrate solution (Absorbance at 450 nm). **(D)** Quantification of the neurite length of differentiated CAD cells (1 × 10^5^ cells/cm^2^) over a 24-well plate after 5, 10, 20, and 40 min of incubation with control medium (without fusion proteins), Syndecan-4-Fc or α_V_β_3_-Fc in serum-free medium, α_V_β_3_-Fc/Protein-A, or α_V_β_3_-Fc/Syndecan-4-Fc (ratio 1:1). **(E)** Quantification of the neurite length of differentiated CAD cells over a 24-well plate (1 × 10^5^ cells/cm^2^) after 40 min incubation with serum-free medium containing α_V_β_3_-Fc (1:10 of the total volume, 100 μl) in the absence or presence of different volumes of serum-free medium containing Syndecan-4-Fc. In **(D,E)** the neurites of at least 100 cells were measured per condition by using NeuronJ plug-in for ImageJ. In all graphs, data are expressed as the mean ± SEM (*n* = 3; *p < 0.05; n.s. non-significant, assessed by Mann–Whitney’s test). In **(D)** *p < 0.05 compared to the control situation at the respective times analyzed.

$$\tau \,\left(F\right)=\tau^{0}\,e^{\left(\frac{x^{‡}\,F}{k_{B}\,T}\right)}$$

was used to describe the effect of force on bond lifetime, where τ^0^ is the lifetime in the absence of force, x^‡^ is the distance to the transition state, F is the force applied to the bond, k_*B*_ is the Boltzmann′s constant, and T is the absolute temperature. The Bell model predicts that the time that both proteins interact decreases exponentially with force (Bell, 1978) and was used due to non-deviation from linearity in the force-dependent lifetime plot. Both force-ramp and constant-force data were processed with Matlab using the “Tweezers Analysis” program developed by Jesse Dill in the laboratories of Carlos Bustamante and Susan Marqusee (Dill, 2012) to estimate rupture force values, lifetime and binding/non-binding event data for each condition evaluated.

### Statistical Analysis

Data are expressed as the mean ± standard error of the mean (SEM) from *n* = 3 or more independent experiments. Results were compared by non-parametric unpaired, one-tailed Mann–Whitney analysis using GraphPad Prism 5 software, as was previously reported (Herrera-Molina et al., 2012; Maldonado et al., 2017). Statistical significance is indicated in each figure.

## Results

### Characterization at the Single-Molecule Level Reveals that Syndecan-4 Increases the Lifetime of the Thy-1–α_V_β_3_ Integrin Binding by Forming a Ternary Complex

Our prior results suggest the existence of a tri-molecular complex in the neuron-astrocyte cell-cell adhesion model, which involves Thy-1, Syndecan-4 and the α_V_β_3_ integrin (Avalos et al., 2009; Kong et al., 2013). Considering that not only the α_V_β_3_ integrin, but also Syndecan-4 acts as a mechano-receptor in other cells (Bellin et al., 2009), we first investigated the mechanical response of these Thy-1-dependent interactions either as bi-molecular binding between Thy-1–Syndecan-4 and Thy-1–α_V_β_3_ integrin, or tri-molecular binding Thy-1–α_V_β_3_ integrin + Syndecan-4. To this end, we purified Fc-tagged recombinant proteins and employed the optical miniTweezers instrument to characterize the interactions between fusion proteins by molecular force spectroscopy (Figure 1A).

We used optical tweezers experiments at a dilution that ensures detection of single binding events to obtain evidence for a direct interaction between Thy-1 and Syndecan-4 (Burgos-Bravo et al., 2018). The two fusion proteins were attached to different size polystyrene beads coated with protein G; Thy-1-Fc was bound to the smaller bead attached to a micropipette, while Syndecan-4-Fc was attached to the bigger bead trapped with a laser beam (Figure 1A). As we previously reported for the Thy-1–α_V_β_3_ integrin interaction, the probability of adhesion between two proteins is estimated from the frequency of adhesion events in a sequence of approaching-retraction cycles between both beads (Burgos-Bravo et al., 2018). Therefore, we performed an adhesion frequency assay using the force-ramp strategy to study binding specificity. Higher adhesion frequencies of Syndecan-4 with Thy-1-Fc wild type [Thy-1(RLD)-Fc] compared to the control conditions with TRAIL-R2-Fc receptor (TRAIL-R2-Fc) or beads without proteins (Buffer) were observed (Figure 1B). TRAIL-R2-Fc is an Fc-tagged protein that serves as a negative control in all our functional assays (Maldonado et al., 2017; Lagos-Cabré et al., 2018).

The Thy-1-Fc mutated in the integrin-binding site [Thy-1(RLE)-Fc] (Hermosilla et al., 2008) showed a similar binding frequency as the wild type Thy-1, ruling out the participation of this domain in the interaction with Syndecan-4 (Figure 1B). Conversely, Thy-1-Fc mutated in the heparin-binding domain [HBD, Thy-1(AEAAA)-Fc] (Avalos et al., 2009; Kong et al., 2013) showed a lower binding frequency with Syndecan-4-Fc (Figure 1B). These results are consistent with the rupture force distribution profiles obtained for each individual interaction, where similar peak rupture forces for the interaction between Syndecan-4-Fc and both Thy-1-Fc wild type and Thy-1(RLE)-Fc were observed (Supplementary Figures S1A,B). As expected, a different rupture force histogram was obtained for the binding of the proteoglycan Syndecan-4 and Thy-1(AEAAA)-Fc mutated in the HBD (Supplementary Figure S1C). These results are indicative of a direct interaction between Syndecan-4 and Thy-1 and the involvement of the Thy-1 HBD in the binding between these two proteins.

A constant-force strategy was implemented with the optical tweezers to characterize the mechanical response of the Thy-1-dependent interactions. Here, once the beads are in contact, the force is quickly ramped to and held at a desired constant force until the interaction is disrupted. Thus, the bond lifetime is determined as the time that it takes to dissociate the bond at a given constant force (Marshall et al., 2003). A representative force-time trace obtained for the interaction between Thy-1-Fc and Syndecan-4-Fc is shown in Figure 1C. Using this methodology, we then measured the bond lifetime for each bi-molecular interaction (Thy-1-Fc–Syndecan-4-Fc and Thy-1-Fc–α_V_β_3_-Fc; Figure 1D) and the tri-molecular interaction (Thy-1-Fc–α_V_β_3_-Fc + Syndecan-4-Fc; Figure 1E) at different constant forces, between 10 and 50 pN, and plotted bond lifetime as a function of constant force. The lifetime data were then fitted by applying the Bell model (Bell, 1978) (see section Materials and Methods). As predicted by the Bell’s equation, Figure 1D shows that force accelerates the dissociation of both bi-molecular interactions, indicative of slip bond behavior. These mechanical responses were corroborated by plotting the force-dependent lifetime data on a logarithmic scale (Supplementary Figure S1D). Using this model, we characterized the kinetic parameters of the unbinding process for each set of bi-molecular binding events in the absence of force (Figure 1F). A significant difference in the mechanical response is observed at forces lower than 10 pN, where the calculated bond lifetime values are lower for the interaction of Thy-1 with the proteoglycan Syndecan-4. The result suggests that this interaction is disrupted more rapidly than Thy-1–α_V_β_3_ integrin binding at forces lower than 10 pN. The higher bond lifetime at zero force that was found for Thy-1–α_V_β_3_ integrin binding (τ^0^ = 6.79 ± 1.49 s) compared to that of Thy-1–Syndecan-4 (τ^0^ = 3.06 ± 0.22 s) suggests that in the absence of mechanical forces, the Thy-1–α_V_β_3_ integrin interaction is more stable than that of Thy-1–Syndecan-4. In addition, a similar distance to the transition state value was characterized for both interactions (∼0.2 nm; Figure 1F), suggesting that despite the differences found in the mechanical responses for each bi-molecular interaction, the nature of these bonds is similar.

We also studied the mechanical response of Thy-1-dependent binding in the presence of both astrocytic proteins, Syndecan-4 and α_V_β_3_ integrin. As a control for binding specificity, we first evaluated the force-dependent lifetime in the presence of the TRAIL-R2-Fc protein (i.e., Thy-1-Fc–Syndecan-4-Fc + TRAIL-R2-Fc and Thy-1-Fc–α_V_β_3_-Fc + TRAIL-R2-Fc; Figure 1E). Under these conditions, the mechanical response and the bond lifetime at zero force were similar to those characterized for each bi-molecular Thy-1–α_V_β_3_ and Thy-1–Syndecan-4 association (Figure 1D). These findings confirm that the mechanical response observed depends on the specific interaction mediated by Thy-1 and its ligands and not on the addition of another component to the system. Interestingly, when the constant-force assay was performed using Thy-1-Fc and α_V_β_3_ integrin + Syndecan-4, we found that force accelerated the dissociation of these molecules (Figure 1E and Supplementary Figure S1E). However, at forces between 0 and 20 pN, the binding lifetime was higher for the tri-molecular complex than for the bi-molecular Thy-1-dependent interactions (Figure 1E). Similarly, the bond lifetime at zero force in the presence of α_V_β_3_ integrin and Syndecan-4 increased to 11.64 ± 1.34 s (Figure 1F), when compared to those τ^0^ values calculated for the bi-molecular binding. These results indicate that both astrocytic proteins, α_V_β_3_ integrin and Syndecan-4, stabilize and enhance binding parameters of Thy-1-dependent interactions in the absence of force.

### Syndecan-4 Accelerates Neurite Retraction Promoted by the α_V_β_3_ Integrin

Our laboratory has shown that the Thy-1–α_V_β_3_ integrin + Syndecan-4 association triggers signals in astrocytes to promote cell movement (Avalos et al., 2009; Kong et al., 2013). On the other hand, α_V_β_3_ integrin binding to Thy-1 in neurons induces neurite retraction (Herrera-Molina et al., 2012; Maldonado et al., 2017). In the latter case, the integrin acts as a ligand rather than a receptor. Thus, we next determined whether the ternary Thy-1–α_V_β_3_ + Syndecan-4 complex acts bi-directionally playing a role in both astrocyte migration and the contraction of the neuronal processes (Figure 2A).

To answer this question, we used Syndecan-4-Fc fusion protein obtained in the supernatant of transiently transfected HEK293T cells (i.e., serum-free medium containing Syndecan-4-Fc; see section “Materials and Methods”). First, we demonstrated the functionality of Syndecan-4-Fc protein: (1) the protein contains heparan sulfate chains as shown by Heparitinase treatment and immunoblot analysis (Figure 2B); (2) Syndecan-4-Fc binds to human basic Fibroblast Growth Factor (bFGF) in an ELISA (Figure 2C), as expected based on the reported existence of an interaction between bFGF and the heparan sulfate chains of Syndecan-4 (Allen et al., 2001; Horowitz et al., 2002). As a negative control, bFGF was incubated with a different Fc-tagged protein (TRAIL-R2-Fc). We observed an increment in the oxidation of the chromogenic substrate TMB, as compared to the other control conditions, when the supernatant containing Syndecan-4-Fc was incubated with bFGF (Figure 2C). These results are indicative of the presence of heparan sulfate chains with ligand binding properties in Syndecan-4-Fc.

We then tested the contribution of Syndecan-4-Fc to neurite retraction induced by α_V_β_3_-Fc integrin. To this end, morphologically differentiated neuron-like CAD cells (i.e., CAD cells with at least 15 μm neuronal processes; see section “Materials and Methods”) were incubated with serum-free HEK293T medium containing the two fusion proteins Syndecan-4-Fc and α_V_β_3_-Fc. Then, morphological changes on CAD cells were recorded (Supplementary Figure S2) and neurite length was quantified (Figure 2D) as described in our previous publications (Herrera-Molina et al., 2012; Maldonado et al., 2017). Neurite retraction reportedly occurs after 20 min of incubation with α_V_β_3_-Fc, and this effect is maintained for at least 40 min of stimulation with the integrin (Maldonado et al., 2017). Thus, the effect of α_V_β_3_-Fc on neurite retraction in the presence of Syndecan-4-Fc at different time points (0–40 min) was tested. When differentiated CAD cells were incubated with Syndecan-4-Fc (inverted purple triangle), neurite length did not change over time (Figure 2D and Supplementary Figure S2). As expected, α_V_β_3_-Fc (green circle) promoted a significant retraction of neurites at 20 and 40 min of incubation. Interestingly, when CAD cells were incubated with α_V_β_3_-Fc and Syndecan-4-Fc (ratio 1:1), a significant reduction in neurite length was observed after 5 min (orange diamond) (Figure 2D and Supplementary Figure S2). One possibility to explain the accelerated neurite contraction is that Syndecan-4 might be facilitating the clustering of integrin and thus, of Thy-1. If this were the case, the addition of Protein A [Protein A favors cross-linking of the Fc-fusion proteins (Moks et al., 1986)] in the absence of Syndecan-4, should induce aggregation of α_V_β_3_-Fc, and thus a similar response. Indeed, we observed that incubation of CAD cells with α_V_β_3_-Fc pre-treated with Protein A induced neurite retraction after already 5 min (blue triangle, Figure 2D), thus accelerating the effect of α_V_β_3_-Fc, just as observed when α_V_β_3_-Fc and Syndecan-4-Fc were added together. We additionally tested the effect of the concentration of Syndecan-4 on the retraction process at 40 min of incubation, by adding α_V_β_3_-Fc and Syndecan-4-Fc at different ratios to differentiated CAD cells. α_V_β_3_-Fc in the absence of Syndecan-4-Fc induced a reduction in neurite length (green bar), compared to the condition without any fusion proteins (gray bar, Figure 2E). Interestingly, neurite retraction promoted by α_V_β_3_-Fc integrin was not significantly modified by the addition of Syndecan-4-Fc at any dilution at this time point (purple bars, Figure 2E). These results agree with those shown in Figure 2D, where no differences in neurite retraction induced by α_V_β_3_-Fc in the presence or absence of Syndecan-4-Fc were found at 40 min. Altogether, the results suggest that although Syndecan-4 interacts directly with Thy-1, recombinant Syndecan-4-Fc by itself has no effect on neuronal CAD cell extensions. However, Syndecan-4 increases the speed at which neurite retraction is promoted by α_V_β_3_-Fc integrin, suggesting that the proteoglycan Syndecan-4 acts as a co-ligand for Thy-1.

### The Inhibitory Effect of Astrocytes on Neurite Extension Requires Syndecan-4

**FIGURE 3:**
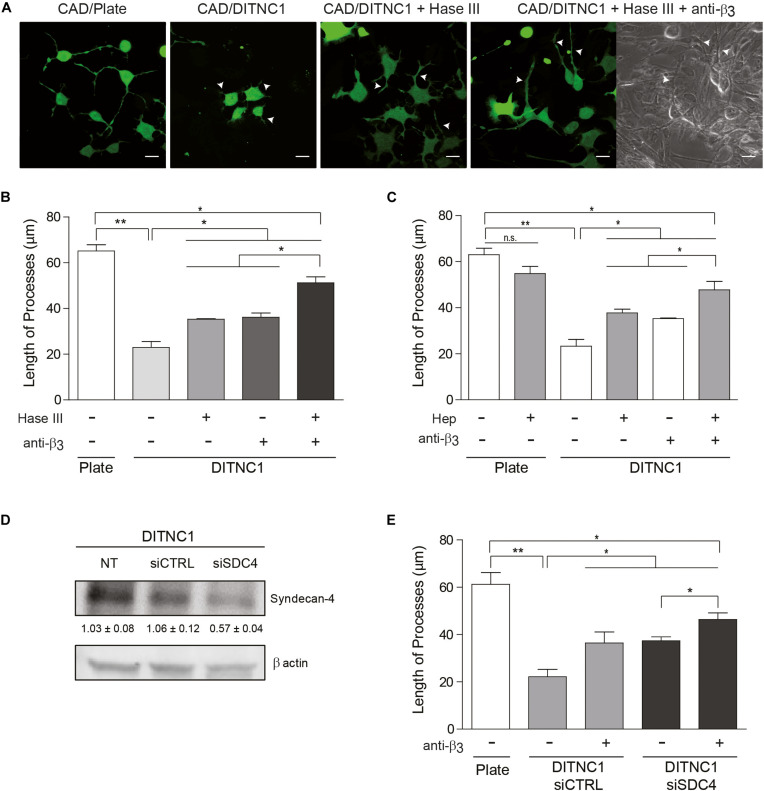
The inhibitory effect of astrocytes on neurite extension requires Syndecan-4. Cell tracker green-labeled CAD cells (5 × 10^4^ cells/cm^2^) were seeded onto a plate or co-cultured on top of a fixed-monolayer of DITNC1 astrocytes. Neurite extension was then induced by serum deprivation for 24 h (1 × 10^5^ cells/cm^2^). To evaluate the participation of heparan sulfate chains in the inhibition of neurite outgrowth, DITNC1 cells were pre-treated with Heparitinase III (Hase III; 0.5 mU; 3 h at 37°C) or pre-incubated with Heparin (Hep; 400 μg/ml; 30 min). To block α_V_β_3_-integrin, astrocytes were incubated with anti-β_3_ integrin antibodies (anti-β_3_; 5 μg/ml; 1 h; 37°C). **(A)** Representative microphotographs of different conditions. Quantification of neurite length (μm) after **(B)** Hase III treatment or **(C)** Hep incubation. **(D)** siRNA silencing of Syndecan-4 protein in whole cell lysates. DITNC1 cells that were either non-transfected (NT), transfected with siRNA control (siCTRL) or with siRNA targeting Syndecan-4 (siSDC4) were evaluated by immunoblotting. Actin was used as a loading control. The band intensities were quantified by ImageJ software and normalized to actin. **(E)** Quantification of neurite length (μm) after Syndecan-4 silencing. For each quantification **(B,C,E)**, neurites of at least 100 cells per condition were evaluated by using NeuronJ plug-in for ImageJ. Arrowheads in **(A)** indicate neurites growing over the DITNC1 astrocytes. In all graphs data are expressed as mean ± SEM (*n* = 3; *p < 0.05; **p < 0.01; n.s. non-significant, assessed by Mann–Whitney’s test).

We next evaluated whether the neuronal response triggered by α_V_β_3_ integrin plus Syndecan-4 also occurs in cell-to-cell communication. As a control, neurite outgrowth was induced in CAD cells seeded in a tissue culture dish. Here, neurons extended processes longer than 60 μm (CAD/Plate) (Figures 3A,B). Such growth was largely inhibited (3-fold) when CAD cells were cultured over a monolayer of fixed DITNC1 astrocytes, which are known to express both α_V_β_3_ integrin (Kong et al., 2013) and Syndecan-4 (Avalos et al., 2009) (CAD/DITNC1) (Figures 3A,B). To test the contribution of Syndecan-4 heparin sulfate groups in neurite outgrowth inhibition, the monolayer of DITNC1 astrocytes was pre-treated with heparitinase III (Hase III) (Levy-Adam et al., 2008). The reduction in neurite extension was not as pronounced when CAD cells were cultured over heparitinase-treated astrocytes (CAD/DITNC1 + Hase III; neurite length ∼35 μm) (Figures 3A,B). Interestingly, treatment with the antibody against β_3_ integrin showed an effect similar to the heparitinase treatment. Then, when the astrocyte monolayer was incubated with heparitinase together with the antibody against β_3_ integrin (CAD/DITNC1 + Hase + anti-β_3_; neurite length ∼52 μm), neurite length increased compared to the condition without any astrocyte pre-treatment (CAD/DITNC1 ∼22 μm) or with individual pre-treatments (∼35 μm, Figures 3A,B). In addition to the length of processes, we characterized morphological differentiation of CAD cells, defined by the presence of at least one neurite of ≥15 μm in length (Supplementary Figure S3A). When CAD cells were seeded onto a tissue culture dish, ∼80% of the cells differentiated. Alternatively, only ∼19% developed such extended processes over a monolayer of DITNC1 astrocytes. However, the percentage of differentiated cells increased after heparitinase treatment (∼50%). As expected, this effect was enhanced when β_3_ integrin was also blocked (∼62%). Another indicator of morphological differentiation is the number of varicosities along CAD processes (Supplementary Figure S3B). Here, results similar to those shown in Figure 3B and Supplementary Figure S3A were obtained. Heparitinase pre-treatment also prevented the reduction of varicosities induced by the astrocyte monolayer. Therefore, the ability of DITNC1 astrocytes to block neurite extension requires Syndecan-4. Importantly, considering that a fixed monolayer of cells was used as a substrate to differentiate the CAD cells, the effects observed can be attributed exclusively to membrane-bound rather than soluble factors.

Considering that Thy-1 interacts directly with sulfated glycans, such as heparin (Hueber et al., 1992), a second strategy was to pre-incubate CAD cells with heparin. In this case, heparin should compete with Syndecan-4 heparan sulfate chains for the Thy-1 HBD. Neurite extension was not affected when CAD cells were pre-incubated with heparin and seeded directly onto a tissue culture dish (CAD + Hep/Plate) (Figure 3C and Supplementary Figure S4); however, inhibition of neurite outgrowth was observed when CAD cells were cultured over astrocytes (CAD/DITNC1) (Figure 3C). In addition, an increase in process outgrowth was observed when CAD cells were pre-incubated with heparin (CAD + Hep/DITNC1), or when the DITNC1 cell monolayer was treated with anti-β_3_ integrin antibodies. These effects were potentiated by the combined addition of heparin and anti-β_3_ integrin antibodies (CAD + Hep/DITNC1 + anti-β_3_) (Figure 3C). The results obtained with heparitinase and heparin treatments suggest that astrocyte heparan sulfate proteoglycans (HSPG) are required to inhibit CAD cell neurite outgrowth induced by Thy-1. To confirm the specific involvement of Syndecan-4 in the neuronal response, we used two different small interference RNA (siRNA) against exons 3 and 4 (Avalos et al., 2009) to silence Syndecan-4 in DITNC1 astrocytes. Immunoblot analysis revealed a 50% reduction in the levels of the proteoglycan after siRNA transfection (siSDC4), compared to the non-transfected (NT) cells or to those transfected with a siRNA control (siCTRL) (Figure 3D). To evaluate whether inhibition of neurite outgrowth depends on astrocytic Syndecan-4, CAD cells were co-cultured over a monolayer of DITNC1 with silenced Syndecan-4, and then neurite extension was induced by serum deprivation. Quantification of neurite length showed a process extension of ∼60 μm in the control condition. In contrast, for DITNC1 astrocytes transfected with siCTRL, neurite length was only ∼21 μm (Figure 3E). This reduction in neurite length was attenuated when β_3_ integrin was blocked with an anti-β_3_ integrin antibody (∼38 μm). A similar effect was observed when CAD cells were seeded over astrocytes transfected with siRNA targeting Syndecan-4 (∼39 μm). Interestingly, when β_3_ integrin was blocked in Syndecan-4 knock down astrocytes, neurite length increased (∼45 μm) (Figure 3E). These findings indicate that inhibition of neurite outgrowth mediated by Thy-1 depends on the combined action of α_V_β_3_ integrin and Syndecan-4 present on the surface of astrocytes.

### Primary Astrocytes Under Pro-inflammatory Conditions Inhibit Neurite Outgrowth in an α_V_β_3_ Integrin- and Syndecan-4-Dependent Manner

**FIGURE 4:**
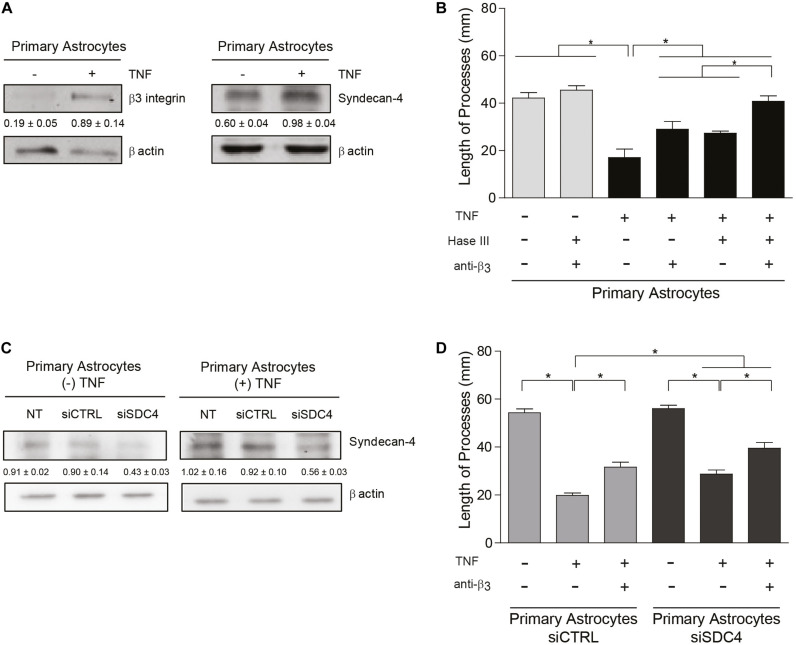
Primary astrocytes under pro-inflammatory conditions inhibit neurite outgrowth in an α_V_β_3_ integrin- and Syndecan-4-dependent manner. **(A,C)** Protein levels of β_3_ integrin and Syndecan-4 in primary astrocytes treated or not with TNF were evaluated by immunoblotting. Actin was used as a loading control. The band intensities were quantified by ImageJ software and normalized to actin. **(B,C)** CAD cells (5 × 10^4^ cells/cm^2^) were seeded on top of a monolayer of primary astrocytes pre-treated or not with TNF (10 ng/ml; 48 h) and neurite extension was induced by serum deprivation. Primary astrocytes were **(B)** pre-treated with Heparitinase (Hase III; 0.5 mU) for 3 h at 37°C or **(D)** transfected with siRNA against Syndecan-4 (siSDC4) to evaluate heparan sulfate chains and Syndecan-4 participation in the inhibition of neurite outgrowth, respectively. To block α_V_β_3_ integrin, astrocytes were also incubated with anti-β_3_ integrin antibodies (5 μg/ml; 1 h; 37°C). **(B,D)** Quantification of neurite length (μm) in co-culture assays. Neurites of at least 100 CAD cells per condition were evaluated by using NeuronJ plug-in for ImageJ. Data are expressed as mean ± SEM (*n* = 3; *p < 0.05, assessed by Mann–Whitney’s test).

Primary astrocytes reportedly display growth promoting properties that favor neurite extension (Hama et al., 2004). Interestingly, astrocytes from adult individuals do not express α_V_β_3_ integrin (Ellison et al., 1998); however, under pathological conditions associated with inflammation, astrocytes adopt a reactive phenotype and upregulate the expression of α_V_β_3_ integrin (Gladson and Cheresh, 1991; Ellison et al., 1998; Del Zoppo and Milner, 2006) and Syndecan-4 (Iseki et al., 2002; Okuyama et al., 2013; Lagos-Cabré et al., 2017). Our previous results indicate that primary astrocytes treated with pro-inflammatory cytokines increment protein levels of α_V_β_3_ integrin and Syndecan-4 *in vitro* and become reactive astrocytes (Lagos-Cabré et al., 2017, 2018). Therefore, we tested the hypothesis that cytokine-treated astrocytes should inhibit neurite outgrowth in a co-culture assay with CAD cells. Immunoblot analysis of primary astrocytes treated with TNF (+TNF) confirmed the presence of elevated levels of α_V_β_3_ integrin and Syndecan-4 in such astrocytes compared to the control condition (−TNF) (Figure 4A). For CAD cells seeded over a monolayer of TNF-treated astrocytes, process extension decreased compared to the control condition without cytokines (Figure 4B and Supplementary Figure S5). Neurite outgrowth under pro-inflammatory conditions (i.e., in the presence of TNF) increased when primary astrocytes were pre-treated with anti-β_3_ integrin antibodies or heparitinase to block Syndecan-4. Moreover, the combined treatment (+Hase; +anti-β_3_) lead to an even greater increase in neurite extension, reaching essentially the same values as in the absence of TNF (Figure 4B and Supplementary Figure S5). To evaluate the specific role of Syndecan-4 on the inhibition of neurite outgrowth, co-culture assays were employed utilizing primary astrocytes in which the proteoglycan Syndecan-4 was silenced. In the absence or presence of TNF, protein levels of Syndecan-4 after transfection with siRNA against Syndecan-4 (siSDC4) remained low compared to the non-transfected (NT) cells or those transfected with an siRNA control (siCTRL) (Figure 4C). CAD cells in serum-free medium grew long processes over primary astrocytes transfected with siCTRL, whereas neurite outgrowth was decreased by the addition of TNF (Figure 4D and Supplementary Figure S6). This inhibition was partially prevented when β_3_-integrin was blocked with specific antibodies (+TNF; +anti-β_3_; siCTRL). On the other hand, when CAD cells were seeded over astrocytes transfected with siSDC4, in the absence of pro-inflammatory conditions (−TNF; siSDC4), neurite length was similar to that observed in the transfection control condition (−TNF; siCTRL). Interestingly, when primary astrocytes transfected with siSDC4 were treated with TNF, extension of neurites was inhibited (+TNF; siSDC4), compared to control condition without TNF (−TNF; siSDC4); however, such inhibition was not as pronounced as that induced by astrocytes transfected with siCTRL under pro-inflammatory conditions (+TNF; siCTRL) (Figure 4D and Supplementary Figure S6). In addition, when β_3_ integrin was blocked in primary astrocytes with silenced Syndecan-4 and treated with TNF, a significant recovery in neurite extension was observed (+TNF; +anti-β_3_; siSDC4). These results indicate that under pro-inflammatory conditions the combined action of the surface molecules α_V_β_3_ integrin and Syndecan-4 in astrocytes promotes inhibition of CAD cell neurite extension. In conclusion, under pathophysiological conditions, where astrocytes are exposed to an inflammatory environment, neurite retraction is promoted in a Syndecan-4-dependent manner.

## Discussion

Signals exchanged between neurons and astrocytes regulate the cytoskeleton of both cells suggesting a bi-directional flow of information between the two cell types (Herrera-Molina et al., 2013; Leyton et al., 2019). In our present study, results obtained at a single molecule level using molecular force spectroscopy indicate that Thy-1 interacts directly with Syndecan-4. We also show that the proteoglycan Syndecan-4 forms a ternary complex with Thy-1 and α_V_β_3_ integrin increasing the Thy-1–α_V_β_3_ integrin bond lifetime, even when exposed to mechanical stress. In a cellular context, the role of Syndecan-4 in this ternary complex is important because it increases the speed of the neuronal responses. This neuronal outcome is facilitated under inflammatory conditions due to the increased expression of Syndecan-4 (Iseki et al., 2002; Properzi et al., 2008) and α_V_β_3_ integrin (Ellison et al., 1998, 1999; Lagos-Cabré et al., 2017).

The ternary molecular complex described here (Thy-1–α_V_β_3_ + Syndecan-4) differs from that reported by Barker’s group (Thy-1–α_5_β_1_ + Syndecan-4) with respect to the integrin heterodimer involved, the biological context, and also the manner in which force regulates bond dissociation (Fiore et al., 2014). The α_5_β_1_ integrin ternary complex formed mediates adhesion between activated endothelium and melanoma cells, while the complex containing α_V_β_3_ integrin is involved in neuron-to-astrocyte bi-directional communication. The bi-molecular Thy-1–α_5_β_1_ integrin bond behaves as a slip bond when force is applied, but when Syndecan-4 is present, a tri-phasic slip-catch-slip behavior is observed implying that force abruptly increases bond strength (Fiore et al., 2014). Although other authors have described a catch bond behavior at forces around 5–10 pN for other ternary complexes, such as that formed by Cadherin-Catenin-Actin (Buckley et al., 2015), Fiore’s findings revealed a catch bond behavior at forces between 20 and 35 pN for the ternary complex. Here, we demonstrate that both bi-molecular complexes formed by Thy-1 (i.e., Thy-1–α_V_β_3_ integrin and Thy-1–Syndecan-4) have slip bond characteristics, as the bond lifetime decreases with forces between 10 and 50 pN (Figure 1D). Also, the absolute values of lifetime in the absence of force suggest a weaker interaction, and therefore a lower relative binding affinity between Thy-1 and Syndecan-4 than with α_V_β_3_ integrin, which might reflect Thy-1 binding to heparan sulfate chains rather than a protein-protein interaction. Indeed, such differences in affinities have also been described for the fibronectin-dependent interactions with either Syndecan-4 or α_5_β_1_ integrin. There too, the binding to the proteoglycan is of lower affinity (Kennelly et al., 2019).

The lifetime values are consistent with the responses in neurons (e.g., neurite retraction) triggered by the astrocyte proteins: α_V_β_3_ integrin induces a significant retraction of neuronal processes, while the proteoglycan Syndecan-4 does not promote any morphological changes (Figure 2D). The slip bond behavior found for both bi-molecular interactions coincides with the mechanical characterization described for Thy-1–α_5_β_1_ binding (Fiore et al., 2014) as well as for FGF with the HSPG (Sevim et al., 2017). Moreover, we found that the ternary complex (Thy-1–α_V_β_3_ integrin + Syndecan-4) is more stable in terms of force-dependent lifetime than each binary complex. The presence of both astrocyte proteins increases the time that Thy-1-dependent interactions last, both in the absence of force (∼11.7 s) as well as when force is applied (e.g., ∼4 s at 20 pN). This extended lifetime is consistent with the enhanced and faster neuronal effect promoted by the α_V_β_3_ integrin in the presence of Syndecan-4, suggesting that the proteoglycan stabilizes the Thy-1–α_V_β_3_ interaction, thereby accelerating its specific biological functions.

In contrast to the mechanical response reported by Barker’s group, the Thy-1–α_V_β_3_ integrin + Syndecan-4 complex dissociates more rapidly under force, indicating that the slip bond properties are retained, and that the nature of the integrin as well as its biological function is what defines how Thy-1-dependent interactions are regulated by external forces. In this context, the α_5_β_1_ integrin determines adhesion strength and is involved in cell adhesion maturation processes (Roca-Cusachs et al., 2012; Fiore et al., 2014). Alternatively, the α_V_β_3_ integrin acts as a mechano-transducer by providing a connection to the cytoskeleton (Roca-Cusachs et al., 2009) and triggering downstream events required for cell migration. Altogether, these findings permit hypothesizing that when Syndecan-4 is present, Thy-1–α_5_β_1_ bond (catch) resists higher forces to form strong and stable adhesion complexes, whereas the Thy-1–α_V_β_3_-bond (slip) is transient and less resistant to mechanical forces facilitating force sensing and mechano-transduction.

In a cellular context, the stimulation of neurons expressing Thy-1 on their surface, with a combination of soluble α_V_β_3_ integrin and Syndecan-4 proteins (α_V_β_3_-Fc and Syndecan-4-Fc), accelerates neurite retraction when compared with stimulation of Thy-1 with only α_V_β_3_ integrin. Interestingly, such accelerated retraction is also seen for neurons stimulated with α_V_β_3_ integrin in a multivalent format (with Protein A) (Herrera-Molina et al., 2012). These results can be explained assuming that Syndecan-4 induces structural changes in α_V_β_3_ integrin to favor more efficient interactions between Thy-1 and the α_V_β_3_ integrin, thereby promoting α_V_β_3_ integrin clustering and activation. In support of this idea, Syndecan-1–α_V_β_3_ integrin interaction reportedly stimulates integrin binding to fibronectin, not only by bringing the two molecules together, but also by inducing integrin activation (Beauvais et al., 2004). Intriguingly, the effect of Syndecan-1 requires that the proteoglycan is ligand-engaged, favoring the possibility that conformational changes and/or clustering of Syndecan-1 are required to induce integrin activation. In this context, the association of the Syndecan-1 ectodomain with α_V_β_3_ integrin provides a docking surface to incorporate the growth factor receptor IGF1R into the complex and promote IGFR1 kinase activity that depends on Syndecan-1 clustering. Then, in turn, activated IGF1R induces changes in the cytoplasmic protein Talin, which promotes integrin activation (Beauvais and Rapraeger, 2010). Therefore, Syndecan-dependent integrin activation is attributable to both molecular clustering and changes in cytoplasmic signaling.

The inhibition of morphological neuronal differentiation induced by the engagement of Thy-1 in cell-cell association requires the collective action of α_V_β_3_ integrin and Syndecan-4 expressed in reactive astrocytes. Accordingly, Syndecan-4 downregulation or the blockade of HS chains with Hase III or Hep, all favor neuronal differentiation and neurite outgrowth. However, when β_3_ integrin is blocked by antibodies, and Syndecan-4 is treated with Hase III or Hep at the same time, neuronal differentiation is promoted to levels where process extension, number of processes, and number of varicosities are similar to, but still significantly lower than those observed in the control samples. These results reveal that although α_V_β_3_ integrin and Syndecan-4 are important for Thy-1-dependent inhibition of neuronal differentiation, additional molecules or the protein core of Syndecan-4 (not affected by Hase III or Hep treatments) likely also contribute to inhibition of neurite outgrowth. Indeed, for CSPGs both the protein core and the chondroitin sulfate chains are known to independently inhibit axonal regeneration (Bandtlow and Zimmermann, 2000; Asher et al., 2001). In contrast, little information is available concerning the regulation and function of astrocytic HSPGs in response to brain pathological conditions. Nevertheless, HS moieties of Syndecan-4 are important to mediate the integrin-independent interaction of Thy-1 with Syndecan-4. This is supported by the optical tweezers results revealing that Thy-1-Syndecan-4 interaction requires the HBD of Thy-1 and does not depend on the presence of the α_V_β_3_ integrin binding domain (RLD) or the occurrence of Thy-1-integrin interaction.

Moreover, HSPGs reportedly act as potential “scaffolds” that bring together two proteins to favor their interaction, and thus regulate where and when signaling events begin (Xu and Esko, 2014). An example of this is the interaction of FGF with its receptor (FGFR), where Syndecan-4 acts as a co-receptor (Carey, 1997). In this case, although the FGF/FGFR binding is of high affinity, the interaction with Syndecan-4 and the subsequent signaling events are amplified in the presence of HS chains (Sperinde and Nugent, 2000). Moreover, the formation of a ternary complex FGF/FGFR/Syndecan-4 has been reported to reduce the dissociation constant (k_off_; or increases the lifetime) of the FGF/FGFR interaction, increase the primary ligand-receptor affinity and favor the activation of FGFR at low concentrations of the ligand (Nugent and Edelman, 1992; Forsten-Williams et al., 2005). This data is consistent with the function established for Syndecan-4 in this study, where we report that the HSPG facilitates neuronal retraction and is required for inhibition of neurite outgrowth mediated by Thy-1–α_V_β_3_ integrin association.

**FIGURE 5:**
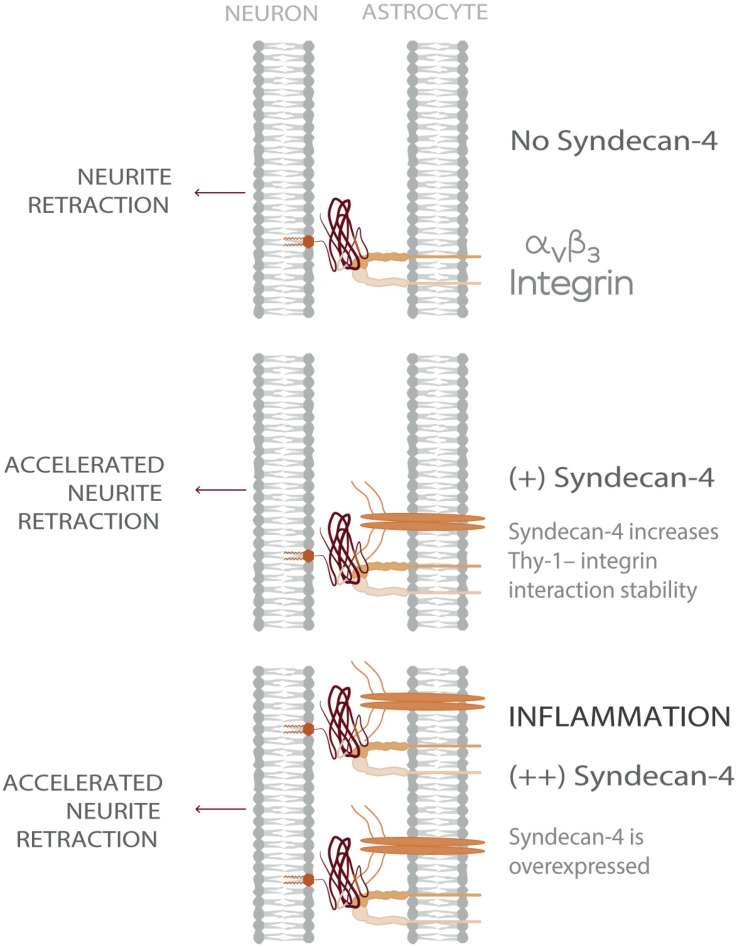
Model showing Syndecan-4 as a key player in neuron-astrocyte interactions under inflammation accelerating axonal retraction. The illustrated behavior is particularly relevant under inflammatory conditions where the expression of both Syndecan-4 and α_V_β_3_ integrin increase on astrocytes and exacerbate the detrimental effects of Thy-1–α_V_β_3_ integrin association, on neurons.

Considering a CNS physio-pathological context, our results suggest that under inflammatory conditions- where both astrocytic α_V_β_3_ and Syndecan-4 protein levels are increased - the ternary complex formed by Thy-1–α_V_β_3_ integrin + Syndecan-4 promotes more stable neuron-astrocyte association (i.e., extended lifetime compared to bi-molecular Thy-1-dependent interactions). Consequently, the effects triggered by these adhesion proteins, such as neurite retraction, are enhanced. However, mechanical cues from the surrounding environment exerted on the Thy-1-α_V_β_3_ integrin + Syndecan-4 complex regulate these cellular processes by stimulating rapid bond dissociation between the molecules. On the other hand, under physiological conditions, when both α_V_β_3_ integrin and Syndecan-4 are expressed at low levels (Lagos-Cabré et al., 2017), bi-molecular interactions are more likely to occur. Thus, the faster bond dissociation of these complexes, with or without mechanical forces, makes stimulation of retraction of neuronal processes unlikely. Therefore, Syndecan-4 appears to play a key role in modulating the speed of neuronal responses under pathological conditions (Figure 5), in agreement with the rapid axon retraction observed *in vivo* (Houle and Tessler, 2003) and *in vitro* (Shao et al., 2019).

Here, we studied morphological neuronal changes stimulated by cell-cell communication between neurons and astrocytes through the cell adhesion molecules, Thy-1, α_V_β_3_ integrin and Syndecan-4. It has also been reported that cell-extracellular matrix interactions play a key role in the regulation of neuronal process retraction (Ahmad et al., 2000; Franze et al., 2009; Shao et al., 2019), as well as in the contraction of other cell types (Okamoto et al., 1998). In our neurite retraction assays, CAD cells were seeded directly over standard tissue culture dishes without additional treatments (e.g., poly-L-lysine). Thus, further investigation is required to decipher how the magnitude of neurite retraction resulting from neuron-to-astrocyte communication is affected by the strength of the adhesion between neurons and the extracellular matrix.

Retraction of neuronal processes is also driven by mechanical tension produced by the active contraction of the actomyosin cytoskeleton (Kranenburg et al., 1997; Govek et al., 2005; Maldonado et al., 2017). Our prior results demonstrate that the engagement of astrocytic α_V_β_3_ integrin with Thy-1 on the surface of neurons triggers specific RhoA-dependent signaling pathways through a preformed membrane complex between Thy-1, the transmembrane protein CBP and the non-receptor tyrosine kinase Src, that drives actomyosin cytoskeleton contraction and, consequently, leads to neurite retraction (Herrera-Molina et al., 2012; Maldonado et al., 2017). Results shown here indicate that Syndecan-4 accelerates the effect of integrin-engaged Thy-1 by interacting directly with the neuronal protein, suggesting that this proteoglycan may also modulate the reported signaling pathway; however, this interesting possibility needs to be confirmed in future experiments.

We describe a Thy-1–α_V_β_3_ + Syndecan-4 ternary complex, which is crucial in determining the Thy-1-dependent response of neurons to astrocytes under inflammation-related diseases in the CNS. In this tri-molecular complex, Syndecan-4 is identified as an enhancer of α_V_β_3_ integrin effect in neurite contractility likely by stabilizing Thy-1–α_V_β_3_ interaction, even when exposed to mechanical forces. Considering that both Syndecan-4 and α_V_β_3_ integrin are upregulated in astrocytes by pro-inflammatory cytokines, our results indicate that under pathological conditions (inflammation), protein expression ultimately determines the formation, as well as the different properties and functions, of the Thy-1–α_V_β_3_ integrin + Syndecan-4 ternary complex (Figure 5). Therefore, our research provides new insights towards understanding how inflammation contributes to neurodegeneration and how mechanical stimuli regulate unbinding properties of adhesion proteins occurring during neuron-to-astrocyte communication.

## Data Availability Statement

All datasets generated for this study are included in the article/Supplementary Material.

## Ethics Statement

The animal use and care protocol was reviewed and approved by Comité de Bioética de Animales, Facultad de Medicina, Universidad de Chile, Protocol #CBA0790-FMUCH.

## Author Contributions

FB-B: conceptualization, investigation, data curation, preparation of all data, figures, and text. SM-M: investigation, methodology, and preparation of figures. AQ: formal data analysis, funding acquisition, and writing—review and editing. CW: visualization, methodology, data curation, formal analysis, and writing—review and editing. LL: conceptualization, formal data analysis, funding acquisition, project administration, work supervision, and writing—review and editing. All authors contributed to the article and approved the submitted version.

## Conflict of Interest

The authors declare that the research was conducted in the absence of any commercial or financial relationships that could be construed as a potential conflict of interest.

## Abbreviations

bFGF: basic fibroblast growth factor
CAD: Cath.a-differentiated
CNS: central nervous system
CSPG: chondroitin sulfate proteoglycans
FA: focal adhesions
FGFR: fibroblast growth factor receptor
Hase III: heparitinase III
HBD: heparan binding domain
Hep: heparin
HS: heparan sulfate
HSPG: heparan sulfate proteoglycans
pN: piconewton
TMB: 3,3′,5,5′-tetramethylbenzidine
TNF: tumor necrosis factor.
