# Supplementary material for: Application of Force to a Syndecan-4 Containing Complex With Thy-1–αVβ3 Integrin Accelerates Neurite Retraction
Source: Front Mol Biosci. 2020 Sep 29;7:582257. doi: 10.3389/fmolb.2020.582257 (PMC7550751; doi:10.3389/fmolb.2020.582257)
Supplement: Supplementary file 1 [file Data_Sheet_1.pdf]

Supplementary Material

Supplementary Figures

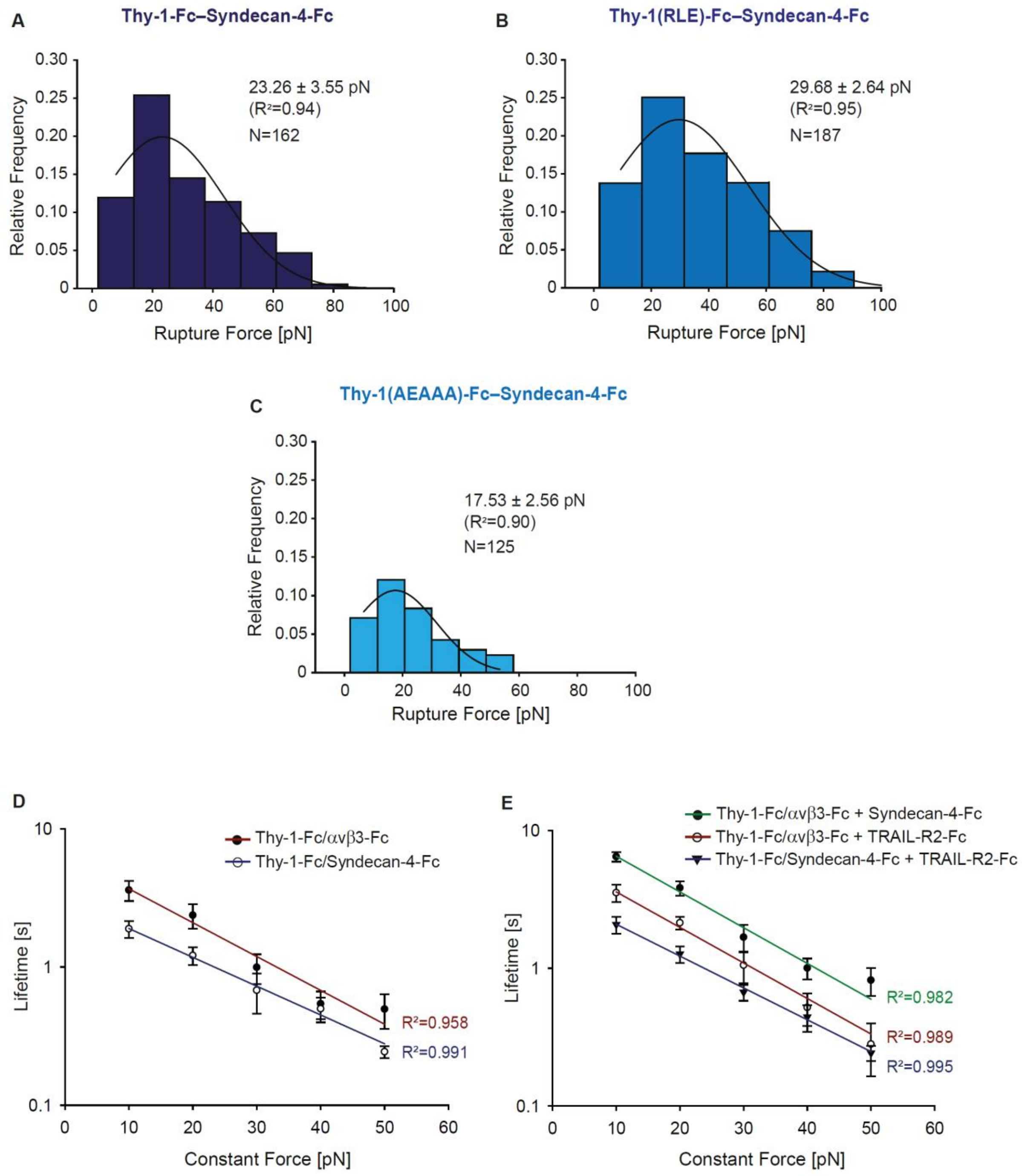

Supplementary Figure 1. Rupture force measurements and force-dependent lifetime for Syndecan-4-Fc interactions with wild type or mutated fusion proteins of Thy-1-Fc. Rupture force

histograms resulting from the interaction between Syndecan-4-Fc with (**A**) wild-type Thy-1-Fc, (**B**) Thy-1(RLE)-Fc mutated in the integrin binding-site, and (**C**) Thy-1-(AEAAA)-Fc mutated in the heparin binding domain (162, 187, and 125 binding event, respectively), and normalized for the total number of approaching-retraction cycles (204, 232, and 318 cycles, respectively) per four pairs of new beads. These rupture force histograms were obtained with force-ramp assays at a loading rate of 10 pN/s. Black lines in each histogram represent the Gaussian distribution curve used to approximate the distribution of rupture forces and calculate each respective peak rupture force. Lifetime of (**D**) bi-molecular or (**E**) tri-molecular Thy-1-Fc interactions plotted on a logarithmic scale as a function of constant force. Adjusted R-squared values are shown.

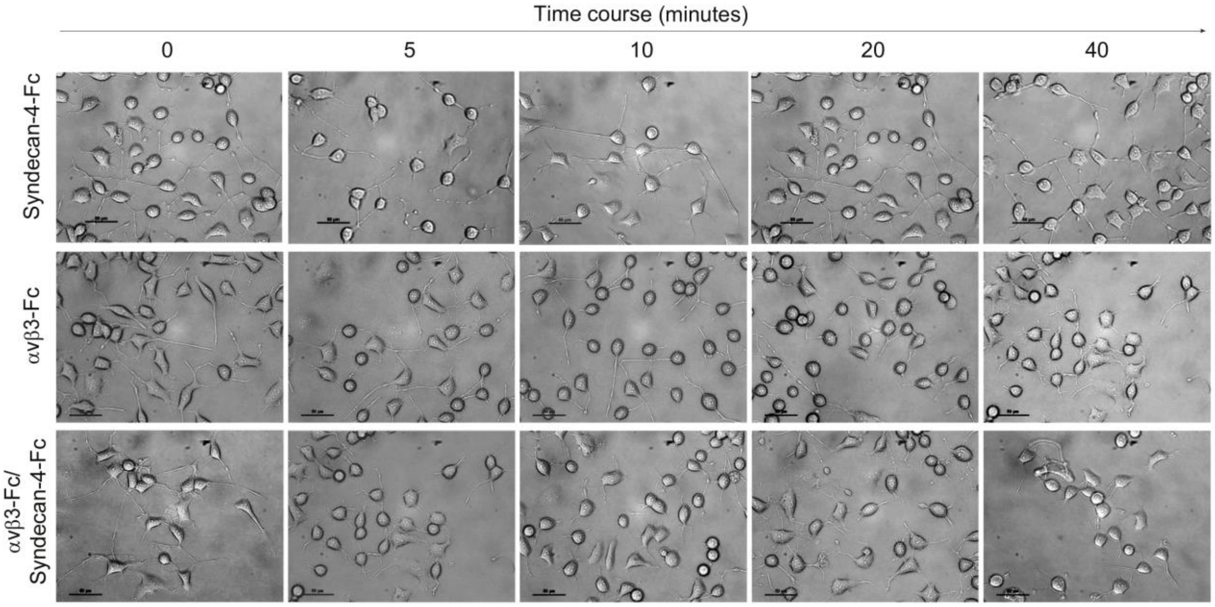

Supplementary Figure 2. Neurite retraction induced by astrocytic αvβ3-Fc and Syndecan-4-Fc fusion proteins. Representative microphotographs of morphologically differentiated CAD cells (time=0) after 5, 10, 20 and 40 minutes of incubation with Syndecan-4-Fc, αvβ3-Fc, or αvβ3-Fc/Syndecan-4-Fc, obtained with phase contrast microscopy and ImageJ software. Scale bar=50 μm.

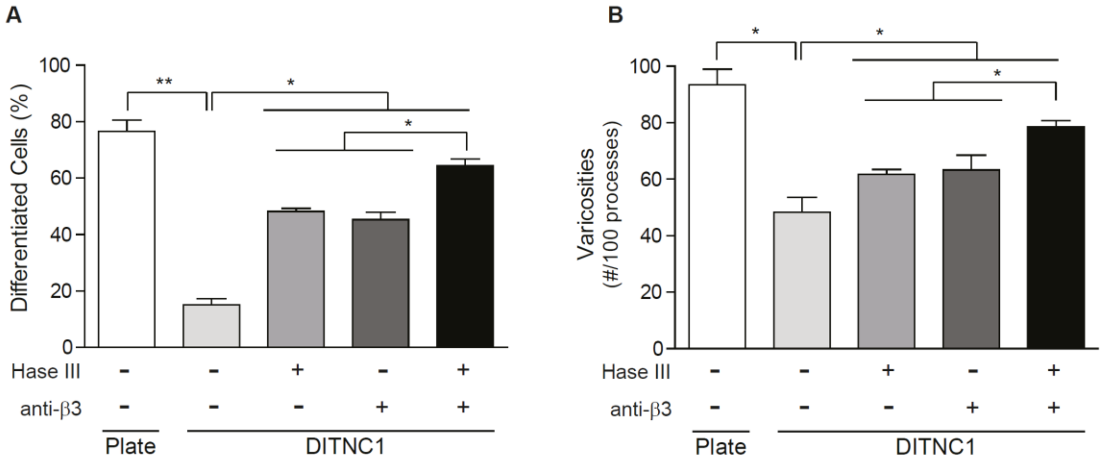

Supplementary Figure 3. Heparitinase treatment precludes the inhibitory effects of DITNC1 astrocytes on CAD cell morphological differentiation. (**A**) Percentage of differentiated CAD cells with at least one neuronal process ≥ 15 µm. For each condition at least 100 cells were evaluated. (**B**) Number of varicosities per 100 processes. Data are expressed as mean ± SEM (n=3; *p<0,05; ** p<0,01, assessed by Mann-Whitney´s test).

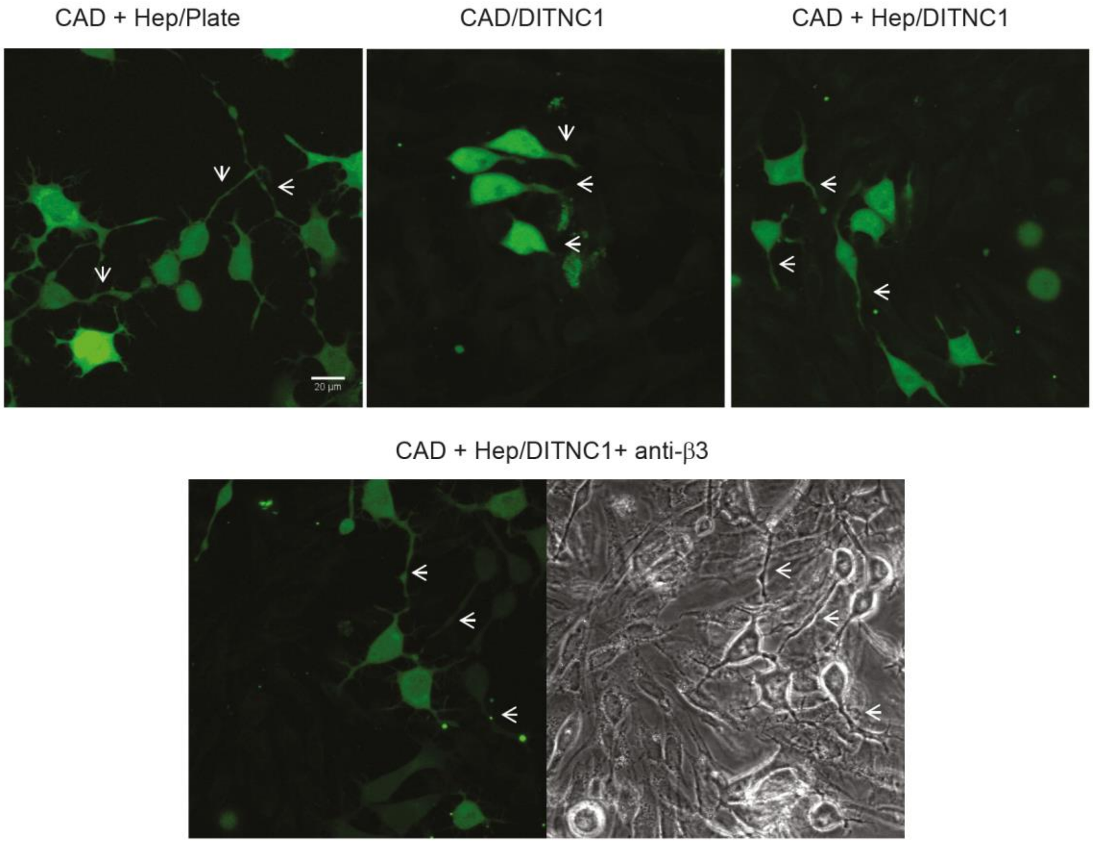

Supplementary Figure 4. Heparin treatment precludes the inhibitory effects of DITNC1 astrocytes on CAD cell neurite outgrowth. Representative microphotographs of neurite (arrows) outgrowth at the indicated conditions, obtained with epifluorescence and phase contrast microscopy and ImageJ software. Scale bar=20 µm.

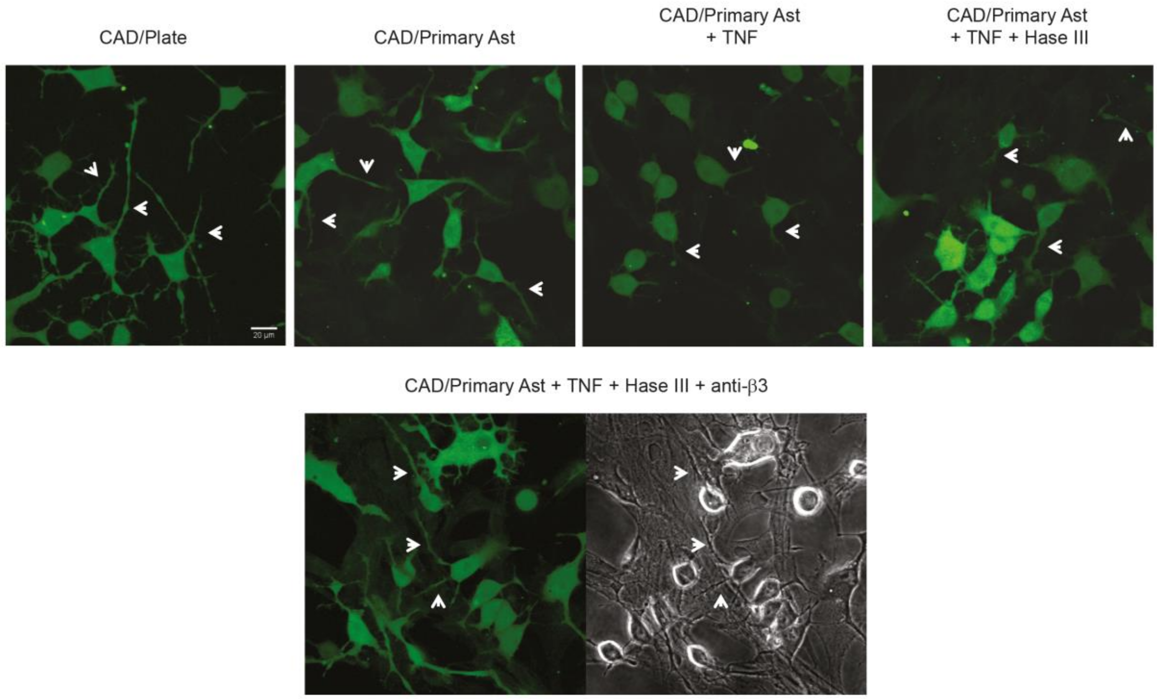

Supplementary Figure 5. Heparitinase treatment precludes the inhibitory effects of primary astrocyte under inflammatory conditions on CAD cell neurite outgrowth. Representative microphotographs of neurite (arrows) outgrowth at different conditions [Heparitinase (Hase III; 0.5 mU) for 3 hours at 37°C and anti-β_3 integrin antibodies (5 µg/ml; 1 hour; 37°C)], obtained with epifluorescence and phase contrast microscopy and ImageJ software. Scale bar= 20 µm.

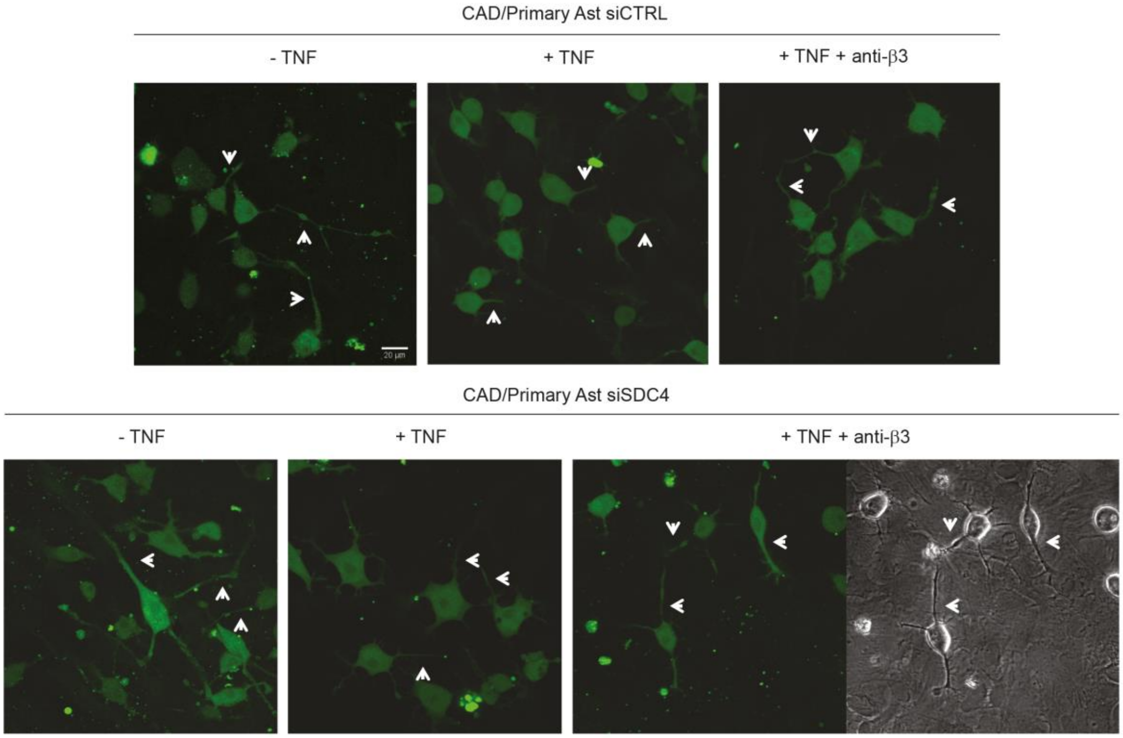

Supplementary Figure 6. Syndecan-4 silencing precludes the inhibitory effects of primary astrocytes under inflammatory conditions on CAD cell neurite outgrowth. Representative microphotographs of neurite (arrows) outgrowth at different conditions [anti-β_3 integrin antibodies (5 µg/ml; 1 hour; 37°C), with silenced Syndecan-4 (siSDC4) or transfected with siRNA control (siCTRL)] obtained with epifluorescence and phase contrast microscopy and ImageJ software. Scale bar= 20 µm.
